# A Parasitoid Wasp Induces Overwintering Behaviour in Its Spider Host

**DOI:** 10.1371/journal.pone.0024628

**Published:** 2011-09-08

**Authors:** Stanislav Korenko, Stano Pekár

**Affiliations:** 1 Department of Agroecology and Biometeorology, Faculty of Agrobiology, Food and Natural Resources, Czech University of Life Sciences, Prague, Suchdol, Czech Republic; 2 Department of Botany and Zoology, Faculty of Sciences, Masaryk University, Brno, Czech Republic; Charité, Campus Benjamin Franklin, Germany

## Abstract

Parasites and parasitoids control behaviors of their hosts. However, the origin of the behavior evoked by the parasitic organism has been rarely identified. It is also not known whether the manipulation is universal or host-specific. Polysphinctine wasps, koinobiont ectoparasitoids of several spider species that manipulate host web-spinning activity for their own protection during pupation, provide an ideal system to reveal the origin of the evoked behavior. Larva of *Zatypota percontatoria* performed species-specific manipulation of theridiid spiders, *Neottiura bimaculata* and *Theridion varians*, shortly before pupation. Parasitized *N. bimaculata* produced a dense web, whereas parasitized *T. varians* built a cupola-like structure. The larva pupated inside of either the dense web or the cupola-like structure. We discovered that unparasitized *N. bimaculata* produce an analogous dense web around their eggsacs and for themselves during winter, while *T. varians* construct an analogous ‘cupola’ only for overwintering. We induced analogous manipulation in unparasitized hosts by altering ambient conditions. We discovered that the behavior evoked by larvae in two hosts was functionally similar. The larva evoked protective behaviors that occur in unparasitized hosts only during specific life-history periods.

## Introduction

Many parasites and parasitoids have evolved remarkable strategies to manipulate the behavior of their hosts in order to promote their own survival and reproduction [1], [2]. The behavioral manipulations described include altered phototaxis, changes in locomotion, and the alteration of foraging and defensive behaviors [2]–[19]. The most fascinating manipulations are those that lead to unnatural host behaviors. The parasitic trematode, *Dicrocoelium dendriticum* Rudolphi, forces its intermediate ant-host to move up onto blades of grass during the night and early morning. This action increases the ingestion of infected ants by grazing sheep, the final host [3]. Mermithid nematodes induce their terrestrial arthropod hosts to commit suicide by jumping into water, after which the hairworms desert the host to spend their adult stage in their natural habitat [8].

Behavioral manipulations often result in the induction of innate behaviors. Acanthocephalan, *Polymorphus paradoxus* (Connell & Corner), evokes evasive behavior in the amphipod intermediate host, *Gammarus lacustris* Sars, which is then eaten by ducks [4]. The braconid parasitoid, *Glyptapanteles* spp., makes their caterpillar host behave as a bodyguard of the parasitoid pupae [15]. The caterpillar stands bent over the parasitoid pupae and violently lashes out at approaching predators, resulting in reduced predation of parasitoid pupae.

Evidence for benefits of the host manipulations for the parasitoid has been gained from several host-parasitoid systems [9]–[12]. But there might be also costs involved. This has been rarely studied. Maure et al. [13] investigated bodyguarding of the braconid pupae, *Dinocampus coccinellae* (Schrank), by ladybird *Coleomegilla maculate* Timberlake. Laboratory experiments revealed that duration of bodyguarding suppressed predation by lacewings but also decreased the parasitoid fecundity.

Within ichneumonid wasps a large group of ectoparasitoids (Polysphinctini) are specialized on spiders [20]. The larva of this koinobiont parasitoids is attached to the dorsal side of the abdomen, where it develops (from egg via three larval instars), while the spider continues foraging. Shortly before pupation the parasitoids manipulate the web-spinning activity of the host in order to establish effective protection against enemies and the environment, though there are a few cases of absent manipulation [21]–[23]. The larva of *Hymenoepimecis* sp. (Ichneumonidae) makes its spider host build a highly modified web that is smaller than the capture web [6], [7], [24]. The protective effect of the web for the parasitoid larva was recently documented by Matsumoto [16], who found that the web structure safely guarded parasitoid larva against predators and scavengers.

The mechanism of manipulation used by parasites or koinobiont parasitoids, i.e. the origin of the induced behavior has not been revealed yet. This is because the host response to manipulation is often very complex; it is composed of a series of behaviors. In this respect, the scenario of wasp parasitoids attacking spiders offers an ideal system for identifying the origin of such induced behavior, because the evoked behavior is simple, i.e. leading to an unique product. Furthermore, koinobiont parasitoids are often not so strictly specialized as idiobionts [25], so the behavioral response can be compared among several hosts.

Here, we chose the wasp *Zatypota percontatoria* Müller (Ichneumonidae), which is specialized on several theridiid spiders. Most frequently it attacks two species, *Neottiura bimaculata* (Linnaeus) and *Theridion varians* Hahn [26]. We performed a comparative analysis of evoked behaviors in two hosts in conditions when these behaviors occur naturally. The obtained results enabled us to identify the origin of the evoked behaviors.

## Results

### Species-specific response

Shortly (24–48 hours) before the larva's pupation the behavior of the host spider changed from typical foraging and web-building activity to the production of a specific structure on the web as detailed below. Once the spider finished the structure, the larva killed the spider, consumed it and built a pupal cocoon inside of the structure.

**Figure 1:**
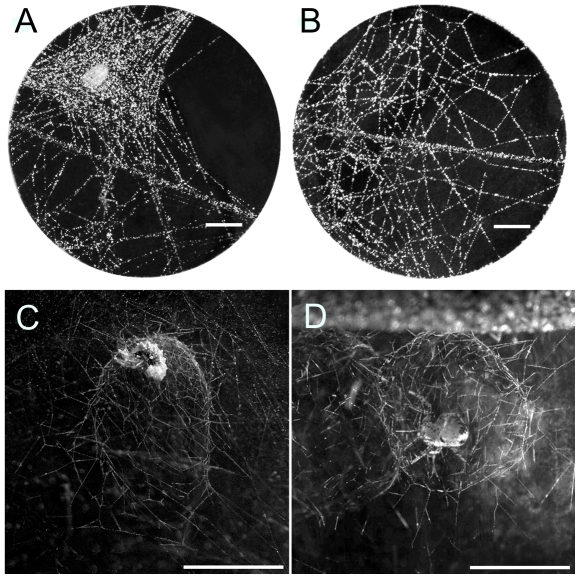
Detail of the web structure in parasitized (A) and unparasitized (B) *N. bimaculata* (dorsal view). Cupola-like structure encloses a wasp pupa with host remnant (C) and an overwintering *T. varians* (D) (lateral view). The wasp larva is in the middle (A, C). Scales: 5 mm.

**Figure 2:**
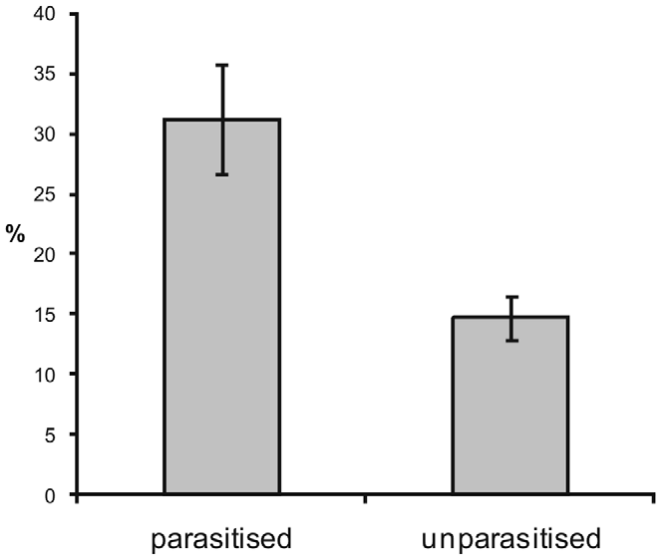
Comparison of the mean density of web in parasitised and unparasitised *N. bimaculata* spiders. Whiskers are standard errors of the mean.

The result of host manipulation induced by the *Z. percontatoria* larva differed dramatically between *N. bimaculata* and *T. varians*. Nearly all parasitized *N. bimaculata* (93%, N = 31) constructed a structure made of dense silk threads surrounding the spider's resting position (Figure 1A). This web was significantly denser (Figure 2) in parasitized *N. bimaculata* than the web of unparasitized individuals (Welch test, *t*_15.1_ = 4.3, *P* = 0.0025, Figure 1B). Most parasitized *T. varians* (82%, N = 56) constructed a closed, spherical cupola-like structure around the spider's resting site in the web (Figure 1C), whereas none of unparasitized spiders constructed such a structure (0%, N = 20) (*X* ^2^_1_ = 38.3, *P*<0.0001). The interior of the ‘cupola’ contained only sparse silk threads. The cupola-like structure was completely closed in 80% of cases (N = 56), and open at the bottom in 20% of cases. The mean horizontal and vertical diameters of the ‘cupola’ were 5.4 mm (SD = 0.06, N = 42) and 6.7 mm (SD = 0.09). The larva pupated either inside the dense web (*N. bimaculata*) or the ‘copula’-like structure (*T. varians*).

### Origin of the evoked behavior

In the field we observed that during winter, unparasitized immature individuals of *T. varians* were hidden in the cupola-like structures built among leaf debris (N = 14) and under tree bark (N = 5). Unparasitized immature individuals of *N. bimaculata* were hidden in a ‘web’ consisting of several threads spread among leaf debris (N = 10).

**Figure 3:**
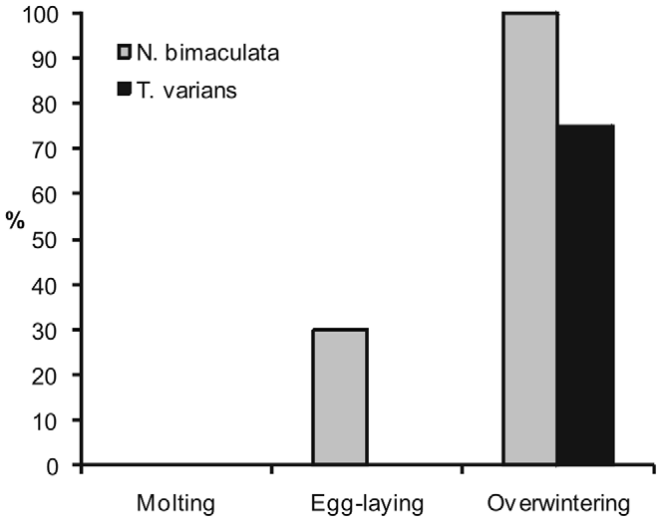
Comparison of the realtive frequency of occurence of dense webs (in case of *N. bimaculata*) or copula-like structure (in case of *T. varians*) at three life-history stages. N varies between 12 and 28 (see Methods for details).

In the laboratory, study of the life history of unparasitized *N. bimaculata* revealed that denser webs were not produced before molting, but during overwintering and around the egg sac (Figure 3). Study of the life history of unparasitized *T. varians* revealed that the spider did not construct the cupola-like structure either before molting or around the egg sac (Figure 3). However, majority of *T. varians* juveniles constructed the cupola-like structure around themselves when exposed to low temperature imitating overwintering (Figure 1D).

## Discussion

We found here that the larva of *Z. percontatoria* manipulated web-spinning activity in two spider hosts. The induced activities were innate to both host species but occurred only during specific life-history periods of unparasitized hosts. In *T. varians* induced activity occurred only before overwintering, while in *N. bimaculata* it occurred both at oviposition and before overwintering. Thus, we identified the origin of the behavior evoked by the parasitic organism. At the same time we revealed that the response of hosts to the parasitoid manipulation was species-specific, though similar in function.

The investigated parasitoid wasp, *Z. percontatoria*, is an example of a parasitoid that attacks a few closely related web-building spiders of the family Theridiidae. All species belong to the ‘*Theridion*’ group, which was recently split into several genera, namely *Theridion*, *Neottiura*, *Phylloneta*, *Platnickina*, *Paidiscura* [27]. These spiders are similar with respect to body size, phenology pattern, and capture web-building [28], [29] but may differ with regard to the construction of defensive structures. We expect that spiders of all these species parasitized by *Z. percontatoria* can produce a variety of silk structures but of similar protective function.

Host manipulation by the polysphinctine parasitic wasp can lead to an unique product that is apparently not produced by unparasitized spiders [23], [24] or it can be a more or less homologous product of a web produced by unparasitized spiders [16], [18], [30]. Manipulated *T. varians* created a cupola-like structure, which was very different from the capture web. The capture web of *T. varians* was an irregular 3-dimensional web about 10 cm across, while the ‘cupola’ was considerably smaller regular 3-dimensional structure. The parasitized spider constructed the ‘cupola’ in the capture web. In nature, the ‘cupola’ is built in the debris among leaves in October and November when the ambient temperature is low. It is used as a shelter by unparasitized spiders during winter. But parasitized spiders construct such a structure in summer on vegetation when temperatures are considerably higher. On the other hand, manipulated *N. bimaculata* produced a protective structure in the capture web by adding more silk around the position for pupation. Such denser silk is used by unparasitized *N. bimaculata* during summer at the time of eggsac production or before overwintering. Again, the parasitized spider constructed such a structure in summer.

Induced changes in host behavior are most likely adaptive as they lead to increased fitness of the parasitoid [13], [31]. In our study system, the behavioral manipulation led to the construction of specific structures that were innate to the spider. We did not study the effect of the structures on the fitness of the parasitoid pupa, but we expect that it is used to increase its survival. The denser web of *N. bimaculata* as well as the cupola-like structure of *T. varians* are assumed to have a similar function for the parasitoid as for the unparasitized spider. The cupola-like structure most likely protects *T. varians* against winter-active predators, and the dense webbing seems to protect eggs of *N. bimaculata* from rain or wind and predators.

Although there are several studies that describe host manipulation by koinobiont parasitoid wasps in detail [1], [5]–[7], [9]–[13], [15]–[19], an understanding of the proximate mechanisms of manipulation is largely lacking. This is in contrast to our knowledge of the mechanism in idiobiont wasp parasitoids, where the wasp injects its venom directly into the host's head ganglia and induces long-term hypokinesia [32], [33]. In koinobionts, the influence of the parasite/parasitoid on host behavior can be either direct by manipulating the host's nervous system, or indirect by manipulating the host's immune or endocrine system, or its metabolism [2], [34]. If the effect is direct, then the behavior is controlled by neuromodulators, such as taurine [35]. Parasites are able to use the same or similar neuromodulators as those of the hosts to usurp control of the host's behavior [2]. However, the production of neuromodulators may be energetically expensive. Less expensive methods may be seen in indirect ways, when parasites induce the host's immune system to produce the appropriate neuromodulators. In such cases the hosts, not the parasites, produce the neuromodulators that alter their own behavior [2], [36].

At present we do not know whether the host behavioural changes observed in our study system was a direct or an indirect influence. The fact that the evoked behavior occurred naturally only during reproduction or overwintering suggests control via the endocrine system. This is also supported by our own induction of the manipulation in unparasitized spiders. By placing unparasitized *T. varians* into cold chambers we induced the production of the cupola-like structure. However, the fact that only a specific short-lived behavioral process was induced rather suggests manipulation via use of neuromodulators. We hypothesize that the larva produced a signal molecule responsible for the onset of such behavior. The molecule should be homologous to a molecule that the spider is naturally producing in conditions requiring protection. Differences in the response of *T. varians* and *N. bimaculata* could be a result of different substances being introduced into different host-species. We are inclined to assume they are a result of species-specific host responses to the same substance (possibly signal molecule) because, in both species, functionally similar behavior was induced.

Parasitism as a life-history strategy among polysphinctine wasps is known for several species. All such species are specialized on spiders from different taxonomical groups and foraging guilds [37]. The more basal genera of the polysphinctine wasps, such as *Clistopyga*, *Dreisbachia*, and *Schizopyga* attack wandering hunters (e.g. Clubionidae, Lycosidae), while more derived genera exploit spiders that construct aerial webs [38]. For example, wasps of the genera *Polysphincta*, *Reclinervellus*, and *Sinarachna* attack orb-web builders from the families Araneidae [37], [39], Nephilidae [19] and Tetragnathidae [6], [7], [17], [24]. Representatives of the genera *Acrodactyla* and *Brachyzapus* attack sheet-web builders from the family Linyphiidae [37] and Agelenidae [16]. Species of the genus *Zatypota* attack space-web builders of the family Theridiidae [18], [26], [30], [37] and Dictynidae [37]. Parasitoids specialized on orb-web builders induce production of a unique simplified web that provides support for the wasp pupa [6], [7], [24], [39]. Parasitoids specialized on sheet-web builders either do not modify host-spinning activity [21] or induce production of webbing for protecting the parasitoid [16]. Parasitoids that attack space-web builders induce production of dense sheet webbing [18]. Thus, the manipulation of web-spinning activity has arisen independently in several lineages within hymenopterans as well as other insect orders. An alteration in spider spinning behavior induced by parasitoid larva has also been observed in acrocerid flies, where the manipulated spider produced a silk cell similar to that made prior to molting [40].

Here, we showed that the parasitoid wasp *Zatypota percontatoria* induced web-spinning activity in both spider hosts. Their response to manipulation was species-specific as parasitized *N. bimaculata* constructed a structure made of dense silk threads and parasitized *T. varians* constructed a cupola-like structure in the web. We revealed the origin of the evoked behavior that the parasitoid used in manipulation. The behaviors were innate to both hosts and occurred only during specific life-history periods of unparasitized hosts, i.e. when spiders needed protection. In *T. varians* such behaviour occurred only before overwintering, while in *N. bimaculata* it occurred both at oviposition and before overwintering. The products of manipulation most likely provided effective protection of the wasp pupa against enemies and the environment.

## Materials and Methods

### Spiders and parasitoids

Parasitized and unparasitized spiders of both species, *Neottiura bimaculata* and *Theridion varians*, were collected in a commercial apple orchard in the Czech Republic, Brno (49° 09′ 37″N, 16° 33′ 35″E) in 2007 and 2008, and in an ecologically managed apple and hazel-nut orchard in Italy, Caraglio, Cascina Rosa (44° 24′ 47.85″N, 7° 24′ 43.39″E) in autumn 2009. Spiders were collected by beating tree branches in spring and in late autumn, when the incidence of parasitism was highest (26). A square shaped beating tray (1 m^2^ area) was placed beneath the tree-crown and all tree branches above the net were beaten. Spiders were kept at a room temperature of 22±3.5°C, natural L∶D regime and fed with a surplus of *Drosophila melanogaster* Meigen flies. Unparasitized spiders were observed until adulthood and parasitized spiders until they were killed and consumed by the parasitoid larva. Once adult parasitoids emerged, they were preserved in ethanol and identified by Kees Zwakhals (Arkel, The Netherlands).

### Host response

We investigated differences in the web structures between parasitized (N = 31) and unparasitized *N. bimaculata* spiders (N = 24) and parasitized (N = 56) and unparasitized (N = 20) *T. varians* spiders. Juvenile spiders collected in the field were placed singly into plastic containers (diameter 34 mm, height 40 mm). When parasitoid larva achieved the last larval instar (2–4 days before the spider was killed) all spiders, including the unparasitized ones, were moved to new plastic containers (of the same size), where spider web construction was observed until the larva had consumed the spider. The web structures of both parasitized and unparasitized spiders were then photographed using a digital camera (Konica Minolta DZ2). The pictures of web structures were analyzed using SigmaScan Pro, v. 5.0. In pictures of *N. bimaculata* webs, the silk density (% of white colour) per selected area that included the center (resting-place) of the web was estimated. In pictures of *T. varians* webs, the size and shape of the cupola-like structures were evaluated.

### Behavior of unparasitized hosts

To reveal whether the manipulated behavior is innate or unique to the two spider species and when it is used, we collected unparasitized specimens of both species and reared them under controlled laboratory conditions with a surplus of *D. melanogaster* flies. Twenty immature *N. bimaculata* individuals and 28 immature *T. varians* individuals were collected in September, placed in plastic containers (as above) and observed during molting. In May we collected 14 adult females of *N. bimaculata* and 12 adult females of *T. varians*, placed them into containers, and observed the web structures until eggsac production. In the orchard we investigated the leaf litter where both spider species overwintered in winter between October 2007 and January 2008 when the average day temperature was 2°C. We recorded the conditions, i.e. microhabitat and type of webbing, in which they were overwintering. The next season, in November 2008, we collected 20 juvenile specimens of *N. bimaculata* and 24 specimens of *T. varians* and placed them into tubes (diameter 34 mm, height 40 mm) with a piece of moist gauze. The tubes were placed into a thermostat at 5°C, L∶D = 10∶14. After 10 days we recorded the frequency and shapes of the constructed web structures.
